# Scoring mechanisms of p16^INK4a ^immunohistochemistry based on either independent nucleic stain or mixed cytoplasmic with nucleic expression can significantly signal to distinguish between endocervical and endometrial adenocarcinomas in a tissue microarray study

**DOI:** 10.1186/1479-5876-7-25

**Published:** 2009-04-14

**Authors:** Chiew-Loon Koo, Lai-Fong Kok, Ming-Yung Lee, Tina S Wu, Ya-Wen Cheng, Jeng-Dong Hsu, Alexandra Ruan, Kuan-Chong Chao, Chih-Ping Han

**Affiliations:** 1 Department of Pathology, Chung Shan Medical University Hospital, Taichung, Taiwan, ROC; 2 Department of Pathology, China Medical University Hospital, Taichung, Taiwan, ROC; 3 Clinical Trial Center, Chung-Shan Medical University Hospital, Taichung, Taiwan, ROC; 4 David Geffen School of Medicine, University of California, Los Angeles. Los Angeles, California, USA; 5 Institute of Medicine, Chung-Shan Medical University, Taichung, Taiwan, ROC; 6 Department of Pathology, School of Medicine, Chung Shan Medical University, Taichung, Taiwan, ROC; 7 Krieger School of Arts and Sciences, Johns Hopkins University, Baltimore, Maryland, USA; 8 Department of Obstetrics and Gynecology, Taipei Veterans General Hospital, and Division of Obstetrics and Gynecology, Faculty of Medicine, National Yang-Ming University School of Medicine, Taipei, Taiwan, ROC; 9 Department of Obstetrics and Gynecology, Chung-Shan Medical University Hospital, Taichung, Taiwan, ROC

## Abstract

**Background:**

Endocervical adenocarcinomas (ECAs) and endometrial adenocarcinomas (EMAs) are malignancies that affect uterus; however, their biological behaviors are quite different. This distinction has clinical significance, because the appropriate therapy may depend on the site of tumor origin. The purpose of this study is to evaluate 3 different scoring mechanisms of p16^INK4a ^immunohistochemical (IHC) staining in distinguishing between primary ECAs and EMAs.

**Methods:**

A tissue microarray (TMA) was constructed using formalin-fixed, paraffin-embedded tissue from hysterectomy specimens, including 14 ECAs and 24 EMAs. Tissue array sections were immunostained with a commercially available antibody of p16^INK4a^. Avidin-biotin complex (ABC) method was used for antigens visualization. The staining intensity and area extent of the IHC reactions was evaluated using the semi-quantitative scoring system. The 3 scoring methods were defined on the bases of the following: (1) independent cytoplasmic staining alone (Method C), (2) independent nucleic staining alone (Method N), and (3) mean of the sum of cytoplasmic score plus nucleic score (Method Mean of C plus N).

**Results:**

Of the 3 scoring mechanisms for p16^INK4a ^expression, Method N and Method Mean of C plus N showed significant (*p-values *< 0.05), but Method C showed non-significant (p = 0.245) frequency differences between ECAs and EMAs. In addition, Method Mean of C plus N had the highest overall accuracy rate (81.6%) for diagnostic distinction among these 3 scoring methods.

**Conclusion:**

According to the data characteristics and test effectiveness in this study, Method N and Method Mean of C plus N can significantly signal to distinguish between ECAs and EMAs; while Method C cannot do. Method Mean of C plus N is the most promising and favorable means among the three scoring mechanisms.

## Background

The histomorphologic overlap of ECA and EMA can make differentiation difficult on H&E in small pre-operative biopsy or curetting specimens. Ascertaining the site of cancer origin may be difficult, but plays an important role in guiding treatment. For the EMA, staging is surgical; however, for the primary ECA, staging is clinical. Treatment protocols may differ substantially between both of them. [1-3]

Previous studies have shown that certain immunohistochemical markers may be helpful in distinguishing between ECAs and EMAs. A traditional 3-marker panel (ER/Vim/CEA) has previously been proposed to make the distinction. A positive ER, Vim and a negative CEA result indicates an EMA; a negative ER, Vim and positive CEA result indicates an ECA. There are, however, many unexpected aberrant immunoexpressions not characteristic of either primary ECAs or EMAs. No study has identified one marker that clearly and consistently makes this distinction in all cases. [4-8]

Recent study has focused on other markers, such as p16^INK4a^, which may express in different intensities, staining patterns and subcellular localizations in various malignancies and tissues. It is also reported that ECAs tends to be positively and diffusely expressed by p16^INK4a^, whereas EMAs tends to be negatively or focally expressed by p16^INK4a ^in routine whole-sectioned tissue slides. [9-13] To date, there is not yet consensus to define the optimal scoring methods of p16^INK4a ^immunoexpression in various tissue samples, especially in those small sizes of pre-operative biopsy or curetting specimens of endocervix or endometrium. In this study, our objective was to propose the appropriately scoring methods and to report that these methods can be easily applied to p16^INK4a ^immunohistochemistry (IHC) as a diagnostic adjunct in distinguishing between ECAs and EMAs. [14-18]

## Materials and methods

### Study materials

The study material consisted of slides and selected formalin-fixed, paraffin-embedded tissue blocks from 38 hysterectomy specimens retrieved from the archives of the Tissue Bank, Clinical Trial Center, Chung-Shan Medical University Hospital. These specimens of known origin, endocervix or endometrium, were accessioned between 2004 and 2008. The cases studied included EMAs (n = 24), as well as ECAs (n = 14). Two board-certified pathologists (CP Han and LF Kok) reviewed all H&E stained slides for these cases. A slide with tumor representative was selected and circled from each case. In the next step, the area corresponding to the selected area on the slide was also circled on the block with an oil marker pen. All these donors' tissue blocks were sent to the Biochiefdom International Co. LTD, Taiwan for tissue microarray construction. They were cored with a 1.5 mm diameter needle and transferred to a recipient paraffin block. The recipient block was sectioned at 5 *u*m, and transferred to silanized glass slides.

### Immunohistochemical staining

Using the Avidin-Biotin Complex (ABC) technique, immunohistochemistry and antigen retrieval methods were applied in the same manner as described in previous literature.[17] Briefly, all the 1.5 mm and 5 *u*m cores of tissue array specimens embedded in paraffin slice on coated slides, were washed in xylene to remove the paraffin, rehydrated through serial dilutions of alcohol, followed by washings with a solution of PBS (pH 7.2). All subsequent washes were buffered via the same protocol. Treated sections were then placed in a citrate buffer (pH 6.0) and heated in a microwave for two 5-minute sessions. The samples were then incubated with a monoclonal anti-mouse p16^INK4a ^antibody (F12, sc-1661, Santa Cruz, 1:200 dilution) for 60 minutes at 25°C. The conventional streptavidin peroxidase method (DAKO, LSAB Kit K675, Copenhagen, Denmark) was performed for signal development and the cells were counter-stained with hematoxylin. Negative controls were obtained by excluding the primary antibody, and positive controls were simultaneously obtained by staining tissues of squamous cell carcinoma of uterine cervix. This slide was mounted with gum for examination and capture by the Olympus BX51 microscopic/DP71 Digital Camera System for study comparison.

### Scoring of IHC staining results

The core of specimens on the tissue microarray (TMA) slides were examined and scored using a two-headed microscope. Because p16^INK4a ^IHC scoring algorithms have not been optimized and standardized, we interpreted the cytoplasmic staining and nucleic staining separately as well as mixed cytoplasmic/nucleic staining collectively. We also adopted the German semi-quantitative scoring system in considering the staining intensity and area extent, which has been widely accepted and used in previous studies. [7-18]Every tumor was given a score according to the intensity of the nucleic or cytoplasmic staining (no staining = 0, weak staining = 1, moderate staining = 2, strong staining = 3) and the extent of stained cells (0% = 0, 1–10% = 1, 11–50% = 2, 51–80% = 3, 81–100% = 4; negative means 0% area staining, focally positive means 1–80% area staining, diffusely positive means 81–100% area staining). The final immunoreactive score was determined by multiplying the intensity scores with the extent of positivity scores of stained cells, with the minimum score of 0 and a maximum score of 12. [19-23]

### Statistical analysis

The threshold for differentiating between final positive and negative immunostaining was set at 4 for interpretation. This optimal cut-off value was determined by using the receiver operating characteristic (ROC) curve analysis (Metz, 1978; Zweig & Campbell, 1993) in this study.[24,25] Score of 4 points or greater was considered positive for p16^INK4a ^expression. A negative stain was classified as having an immunostaining score of 0 to 3 (essentially negative) and indicated a diagnosis of an EMA; whereas a positive stain was classified as having an immunostaining score of 4 to 12 (at least moderately positive in at least 11–50% of cells) and indicated a diagnosis of an ECA. A chi-squared or Fisher's exact test was performed to test the frequency difference of p16^INK4a ^immunostaining (positive vs. negative) between groups of two primary adenocarcinomas (ECAs *vs *EMAs). A nonparametric analysis of Mann-Whitney U-test was used to test the immunostaining raw scores between the two adenocarcinomas, given the fact that the analytical IHC scores were not normally distributed. In addition, we also examined associations among the 3 different scoring mechanisms, based on the subcellular localizations of p16^INK4a ^expression, including (1) Method C, (2) Method N, and (3) Method Mean of C plus N. The nonparametric Spearman's rho correlation coefficient was used to analyze associations between pairs of these three types of p16^INK4a ^scores. Data were analyzed using standard statistical software (SPSS, Inc., Chicago, IL). All tests were 2-sided and the significance level was 0.05.

To evaluate and compare the patterns of p16^INK4a ^expression in making a diagnostic distinction of primary ECAs from primary EMAs, the sensitivity, specificity, accuracy, and the positive and negative predictive values (PPV and NPV respectively) were compared and displayed. Sensitivity is defined as the probability of positive p16^INK4a ^stain in primary ECAs. Specificity is, on the other hand, defined, as the probability of negative p16^INK4a ^stain in primary EMAs.^18 ^Overall accuracy is the proportion of true diagnosis of ECAs and EMAs in total number of p16^INK4a ^scoring tests. Positive predictive value is the probability that a patient with a positive p16^INK4a ^expression has a primary adenocarcinoma of endocervical origin. Negative predictive value is the probability that a person with a negative p16^INK4a ^expression has a primary adenocarcinoma of endometrial origin.[26] In order to assess whether the test results were statistically different from each other based on correct diagnosis, McNemar's test was performed. A *p-value *< 0.05 was considered significant.

## Results

**Figure 1:**
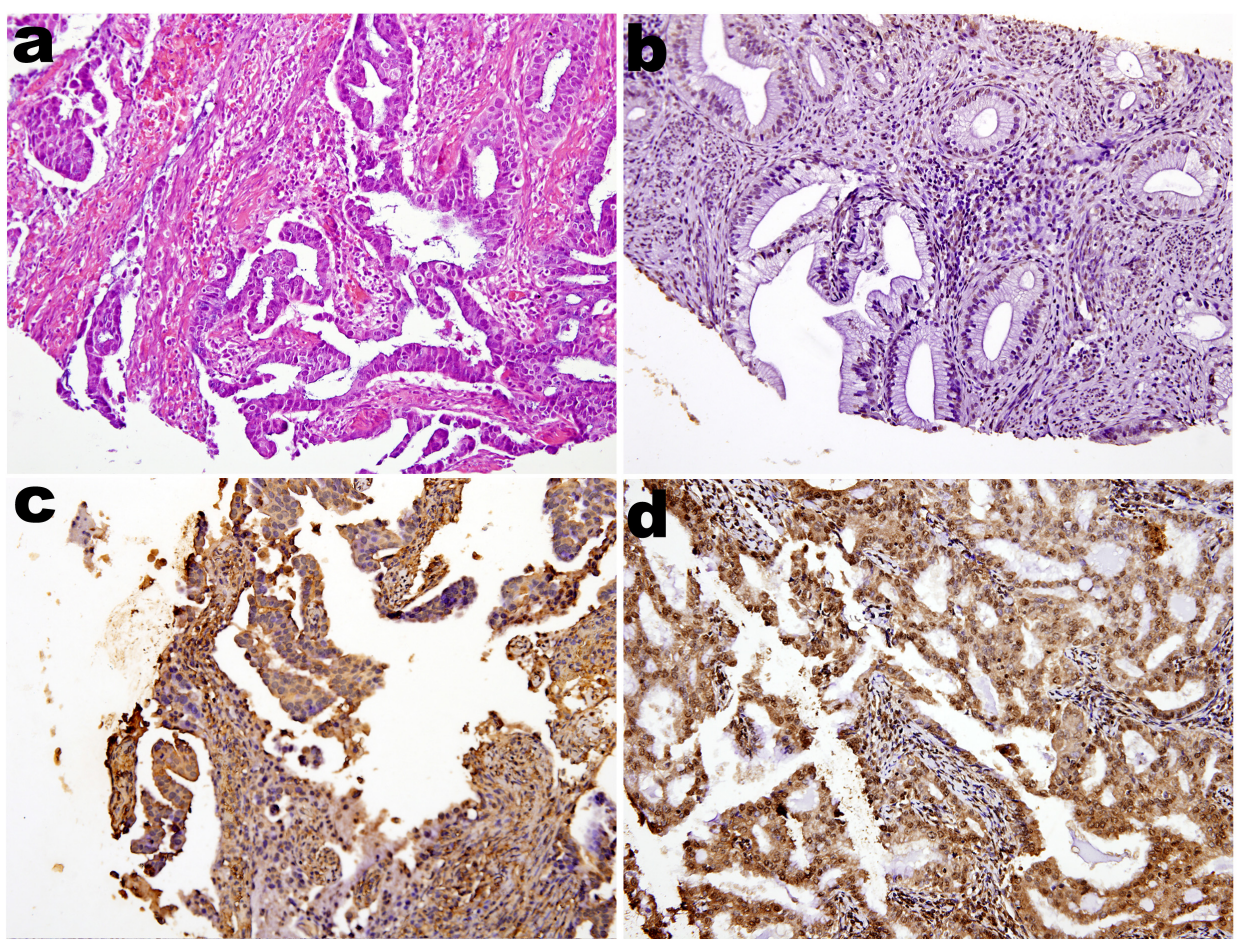
**Immunohistochemical analysis of p16^INK4a ^staining in endocervical adenocarcinomas**. (**a) **Photomicrograph revealed adenocarcinoma of endocervix, endocervical type, H&E stain. **(b) **Photomicrograph revealed tumor with more predominant p16^INK4a ^staining at nuclei than that at cytoplasms. Focally moderately positive nucleic staining and no cytoplasmic staining were identified. **(c) **Photomicrograph revealed tumor with more predominant p16^INK4a ^staining at cytoplasms than that at nuclei. Diffusely moderately positive cytoplasmic staining and focally weakly nucleic staining were identified. **(d) **Photomicrograph revealed tumor with dual prdominat p16^INK4a ^staining at both cytoplasms and nuclei. Diffusely strongly positive nucleic staining and cytoplasmic staining were identified. All photomicrographs **a**, **b**, **c**, **d **were taken in median-powered, ×200

**Figure 2:**
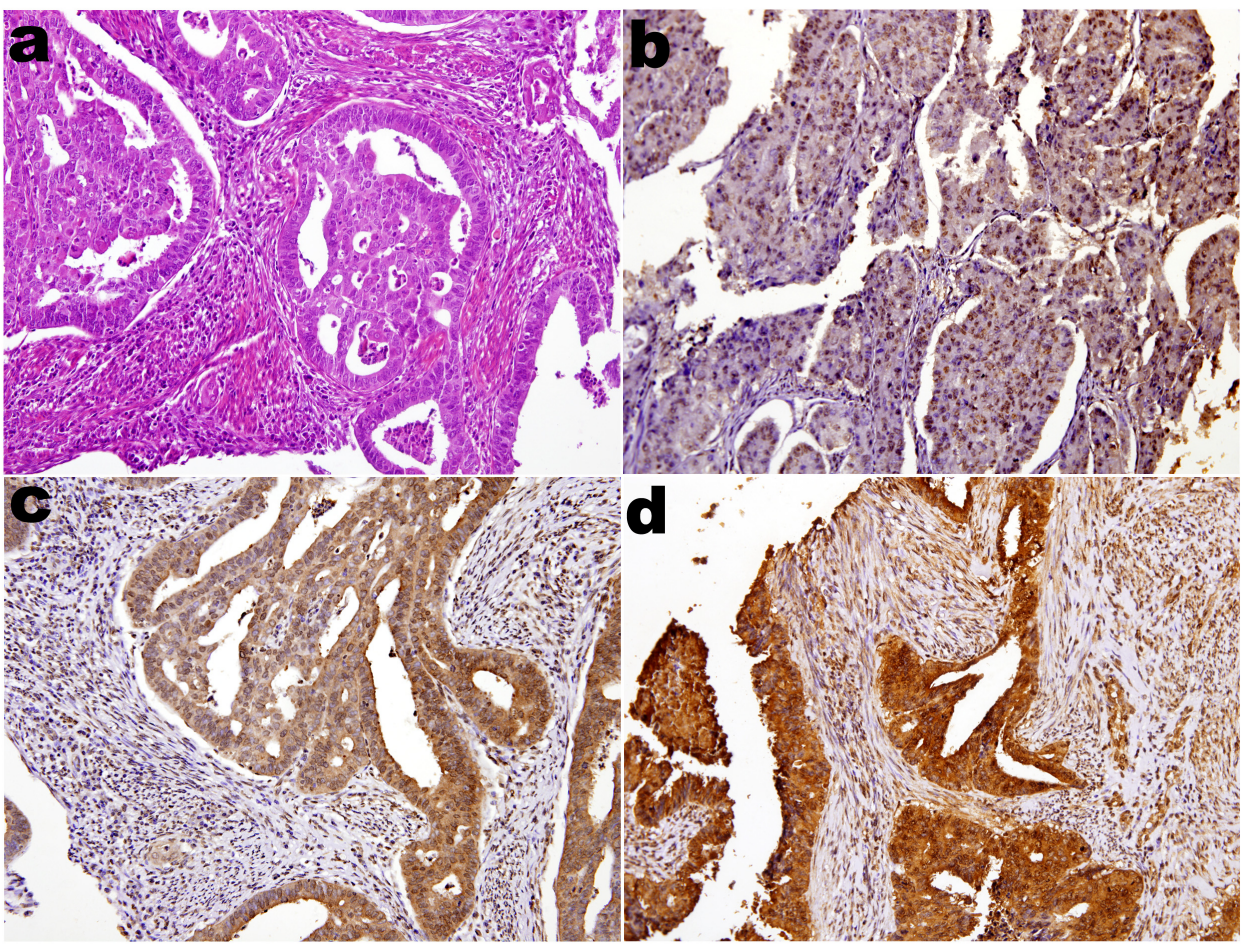
**Immunohistochemical analysis of p16^INK4a ^staining in endometrial adenocarcinomas**. (**a**) Photomicrograph revealed adenocarcinoma of endometrium, endometroid type, H&E stain. **(b) **Photomicrograph revealed tumor with more predominant p16^INK4a ^staining at nuclei than that at cytoplasms. Diffusely moderately positive nucleic staining and no cytoplasmic staining were identified. **(c) **Photomicrograph revealed tumor with more predominant p16^INK4a ^staining at cytoplasms than that at nuclei. Diffusely moderately positive cytoplasmic staining and focally weakly nucleic staining were identified. **(d) **Photomicrograph revealed tumor with dual prdominat p16^INK4a ^staining at both cytoplasms and nuclei. Diffusely strongly positive cytoplasmic staining and nucleic staining were identified. All photomicrographs **a**, **b**, **c**, **d **were taken in median-powered, ×200.

For evaluation of p16^INK4a ^immunohistochemistry, nucleic and cytoplasmic stains were taken into account separately as well as collectively for all cases. H&E (Figure 1a and Figure 2a) and immunoreactivities for p16^INK4a ^can be identified in representatives of ECAs (Figure 1b, 1c and 1d) and EMAs (Figure 2b, 2c and 2d). The p16^INK4a ^expression in ECAs was observed both in nuclei and cytoplasms with varying degrees of staining intensity and area extent. Nucleic stains were predominant in 7 out of 14 cases (Figure 1b), cytoplasmic stains were predominant in 2 out of 14 cases (Figure 1c), while both nucleic and cytoplasmic stains were co-dominant in 5 out of 14 cases (Figure 1d). On the other hand, the p16^INK4a ^expression in EMAs was also observed both in nuclei and cytoplasms with varying degrees of staining intensity and area extents, except for 4 out of 24 cases with a score of 0. Nucleic stains were predominant in 8 out of 24 cases (Figure 2b), cytoplasmic stains were predominant in 6 out of 24 cases (Figure 2c), while both nucleic and cytoplasmic stains were co-dominant in 6 out of 24 cases (Figure 2d).

The IHC results of these three p16^INK4a ^scoring mechanisms, (1) Method C, (2) Method N, (3) Method Mean of C plus N, are summarized in Table 1. By using score of 4 as a cut-off point, except for Method C, the other two scoring mechanisms based on N, and Mean of C plus N, showed significant frequency differences between immunostaining (positive vs. negative) in tissues from the two adenocarcinomas (ECA vs. EMA) in origin. Individually, (1) Method C stained positive in 5 out of 14 (35.7%) ECA tumors and 4 out of 24 (16.7%) stained positive in EMA tumors (p = 0.245), with median staining score and range of 2 (0–12) and 2 (0–12), respectively (p = 0.152); (2) Method N stained positive in 11 out of 14 (78.6%) ECA tumors and 7 out of 24 (29.2%) stained positive in EMA tumors (p < 0.001), with median staining score and range of 5 (2–12) and 2 (0–9), respectively (p < 0.001); (3) Method Mean of C plus N stained positively in 10 out of 14 (71.4%) ECA tumors and 3 out of 24 (12.5%) stained positively in EMA tumors (p < 0.001), with median staining score and range of 4.25 (2–12) and 2 (0–10.5), respectively (p < 0.001). In summary, Method C did not show statistically significant, whereas Method N and Method Mean of C plus N revealed statistically significant frequency differences (p < 0.05) in distinguishing between ECAs and EMAs.

**Table 1:** Scoring methods based on p16^INK4a ^expression patterns and subcellular loci in ECA and EMA

Scoring Method	Score categories	ECA	EMA	*p-value*
C	Score 0–3	9 (64.3%)	20 (83.3%)	.245^a^
	Score 4–12	5 (35.7%)	4 (16.7%)	
	Median (Range)	2 (0–12)	2 (0–12)	.152
N	Score 0–3	3 (21.4%)	17 (70.8%)	.006^a^
	Score 4–12	11 (78.6%)	7 (29.2%)	
	Median (Range)	5 (2–12)	2 (0–9)	<0.001^b^
Mean of C plus N	Score 0–3	4 (28.6%)	21 (87.5%)	<0.001^a^
	Score 4–12	10 (71.4%)	3 (12.5%)	
	Median (Range)	4.25 (2–12)	2 (0–10.5)	<0.001^b^

PS:

1. C: Method of scoring based on independent cytoplasmic staining alone, irrespective of nucleic staining.

2. N: Method of scoring based on independent nucleic staining alone, irrespective cytoplasmic staining.

3. Mean of C plus N: Method of scoring based on mean of cytoplasmic score plus nucleic score.

Note:

• The "a" is Chi-squared test with continuity correction or Fisher exact test, the "b" is Mann-Whitney U test using exact significant.

•Using score 4 points as a cutoff, the immunostains are defined "negative" for scores from 0 to 3, and "positive" for scores from 4 to 12 points.

**Figure 3:**
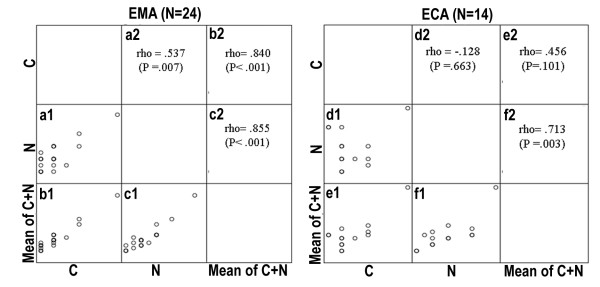
**Scatter plots showing the Spearman's rho correlation coefficients (p value) for the associations between pairs of these three types of p16^INK4a ^scoring mechanisms in endocervical adenocarcinomas and endometrial adenocarcinomas**. (1) **a1/a2 and d1/d2**: Method C was positively correlated with Method N in EMAs, but was not in ECAs. (2) **b1/b2 and e1/e2**: Method C was positively correlated with Method Mean of C plus N in EMAs but was not in ECAs. (3) **c1/c2 and f1/f2**: Method N was positively correlated with Method Mean of C plus N in both EMAs and in ECAs.

The associations between these three scoring methods in ECAs and EMAs were also explored and shown in Figure 3. The immunostaining scores based on Method C were significantly positive correlated with those based on Method N in EMAs (Figure 3 a1 and a2, Spearman's rho = 0.537, p = 0.007), but the correlation was non-significant in ECAs (Figure 3 d1 and d2, Spearman's rho = -0.128, p = 0.663). Method C scores also exhibited significant positive correlation with Method Mean of C plus N scores (Figure 3 b1 and b2, Spearman's rho = 0.840, p < 0.001) in EMAs but did not exhibit significant positive correlation in ECAs (Figure 3 e1 and e2, Spearman's rho = 0.456, p = 0.101). Moreover, Method N scores exhibited significant positive correlation with Method Mean of C plus N scores in both EMAs (Figure 3 c1 and c2, Spearman's rho = 0.855, p < 0.001) and ECAs (Figure 3 f1 and f2, Spearman's rho = 0.713, p = 0.003).

Clinicians may also find interesting the following parameters when judging the test effectiveness of p16^INK4a ^expression as a marker for diagnostic distinction between ECAs and EMAs. Table 2 shows the diagnostic performance of these three different scoring mechanisms for measuring p16^INK4a ^expression in distinguishing 14 ECAs from 24 EMAs. (1) When using Method C, the sensitivity of positively stained ECAs was 35.7% (5/14) and PPV was 55.6%, whereas the specificity of negatively stained EMAs was 83.3% (20/24) and NPV was 69.0%. The overall accuracy rate was 65.8%. (2) When using Method N, the sensitivity was 78.6% (11/14) and PPV was 61.1%, whereas the specificity was 70.8% (17/24) and NPV was 85%. The overall accuracy rate was 71.4%. (3) When using Method Mean of C plus N, the sensitivity was 71.4% (10/14) and PPV was 76.9%, whereas the specificity (21/24) was 87.5% and NPV was 84.0%. The overall accuracy rate was 81.6%, the highest among the three scoring methods (table 2). Furthermore, the 95% confidence intervals (CIs) of these performance parameters were calculated and provided in Table 2 for these three scoring methods. It was clearly that the 95% CI of the sensitivity value for Method C did not overlap with Method N, and Method Mean of C plus N as well. This implied that the performances of sensitivities were different among these three scoring methods especially for Method C. To confirm this finding, McNemar's test was further used to compare the positive rate of p16^INK4a ^expression for the three scoring methods. It demonstrated that the positive rates between Method C and Method N were statistically significantly different (p = 0.022), although non-significances were seen in Method C *vs. *Method Mean of C plus N (p = 0.219) as well as Method N *vs. *Method Mean of C plus N (p = 0.125).

**Table 2:** Diagnostic performance of 4 scoring methods for measuring p16^INK4a ^expression in correctively distinguishing 14 ECA from 24 EMA

Scoring method	C	N	Mean of C plus N
Sensitivity	35.7%	78.6%	71.4%
(95% CI)	(20.5%,50.9%)	(65.5%,91.6%)	(57.1%,85.8%)
Specificity	83.3%	70.8%	87.5%
(95% CI)	(71.5%,95.2%)	(56.4%,85.3%)	(77.0%,98.0%)
PPV	55.6%	61.1%	76.9%
(95% CI)	(39.8%,71.4%)	(45.6%,76.6%)	(63.5%,90.3%)
NPV	69.0%	85.0%	84.0%
(95% CI)	(54.3%,83.7%)	(73.6%,96.4%)	(72.3%,95.7%)
Accuracy	65.8%	73.7%	81.6%
(95% CI)	(50.7%,80.9%)	(59.7%,87.7%)	(69.3%,93.9%)

PS:

1. C: Method of scoring based on independent cytoplasmic staining alone, irrespective of nucleic staining.

2. N: Method of scoring based on independent nucleic staining alone, irrespective cytoplasmic staining.

3. Mean of C plus N: Method of scoring based on mean of cytoplasmic score plus nucleic score.

Note:

Negative p16^INK4a ^expression (score 0–3) tends to be EMA, whereas positive p16^INK4a ^expression (scores 4–12) tends to be ECA.

## Discussion

Distinguishing between ECAs and EMAs before planning the patient treatment is clinically important. When the tumor involves both the uterine endometrium and the endocervix, it becomes difficult to distinguish the primary site of the tumor during preoperative assessment of the limited sizes of biopsy or curetting specimens. In this study, we evaluate various p16^INK4a ^expression patterns in both ECAs and EMAs. We also investigate the most appropriate and effective p16^INK4a ^IHC scoring methods in distinguishing these two types of gynecologic cancers in Taiwanese women. Our valuable domestic data can be extrapolated to women in general and will be helpful in referring and managing such cases worldwide.

The p16^INK4a ^(cyclin-dependent kinase inhibitor 4) is a tumor suppressor protein that binds to cyclin-cdk4/6 complexes, which blocks kinase activity and inhibits progression to the S phase of the cell cycle in the nucleus.[10,15,27-35] However, interpretation of IHC data of p16^INK4a ^staining results is complicated because of its unclear biological significance of cytoplasmic staining and lack of universal accepted algorithm in scoring methodology. Cytoplasmic reactivity is often regarded as unexpected, unspecific event.[36] Some consider only nucleic p16^INK4a ^labeling in tumor cells to be positive and ignore cytoplasmic staining.[16,32] Others state that both nucleic and cytoplasmic immunoreactivities in tumor cells are characteristic and are indeed due to p16^INK4a ^expression. [24-28] It has also been reported that strong cytoplasmic staining in mammary carcinomas is associated with negative prognostic factors, such as low differentiation, p53, Ki-67 labeling etc. We have learned that despite nucleic expression, p53 tumor suppressor protein is localized on cell cytoplasm, where it is regarded as a way of functional inactivation. [29-31] From our data, we cannot draw any conclusion yet about the biological significance of cytoplasmic p16^INK4a ^expression. The knowledge about the functional meaning of cytoplasmic p16^INK4a ^expression is still limited and further large-scale studies are encouraged in various human tissues and tumors.

There are a variety of IHC scoring methods including computer-based plans in literatures, and still seems to be no generally accepted protocols in research laboratories and clinical practices for rating and scoring the immunostaining results. Comparing commercially derived computer-based programs with the conventional analyses by pathologists, there are still lacks of optimized and standardized IHC scoring algorithms. As a result, the objective accuracy did not significantly improve clinical outcome measures. [33-36]

There are also various quantitative scoring mechanisms of p16^INK4a ^expression using various cut-off thresholds in literature. Without mentioning the grading of intensity, Vallmanya Llena FR reports the cut-off point for p16^INK4a ^expression to be 15% positively staining extent.[37] Fregonesi PA defines the cut-off point for p16^INK4a ^expression to be 5% cells stained positively. [32] Khoury T used the positive staining area >50% as a cut-off.[16] They all took both nucleic and cytoplasmic p16^INK4a ^IHC staining into considerations. However, Huang HY regarded any nucleic labeling of p16^INK4a ^to be positive, irrespective of cytoplasmic staining.[38] Kommoss S only used the nucleic staining patterns for p16^INK4a ^evaluation.[39] Milde-Langosch K defined the 12-tier scoring system which was also used in this study.[40] In addition, we investigated the three p16^INK4a ^IHC scoring mechanisms and determined the most effective means in the distinction between ECAs and EMAs. These results can potentially be applied to future clinically diagnostic techniques, when using p16^INK4a^immunohistochemistry.

McCluggage WG (2003) and Mittal K (2007) stated that a diffuse, strong staining pattern of p16^INK4a^, involving nearly all tumor cells tends to be an ECA, whereas, focal, patchy staining pattern of p16^INK4a ^involving 0–50% of cells tends to be an EMA in routine whole-sectioned tissue slides.[15,17] We did not use the patchy or diffuse pattern of p16^INK4a ^IHC staining as a diagnostically distinctive criterion between ECAs and EMAs in this TMA study, because we think that cases with primary EMA may seem to over-express p16^INK4a ^beyond the limited 1.5 mm core area and therefore mimic a diffuse pattern of ECA primary. Instead, we preferred to use the semi-quantitative scoring system in considering the 0–3 points of staining intensity and 0–4 points of staining area extent by multiplying both, yielding a range of score 0 to 12 points. We then divided the results by an appropriate cut-off threshold of 4 to a two-tier of negative (0–3 points) or positive (4–12 points) for interpretation. The mixed cytoplasmic and nucleic stains with varying degrees of intensity and extent in the same tissue section were not uncommon. These discrepancies of p16^INK4a ^expression in different subcellular compartments (cytoplasmic *vs. *nucleic) may have caused significant difficulties in the scoring process.

In this study, we defined 3 scoring mechanisms of the p16^INK4a ^IHC expressions, as follows: (1) independent cytoplasmic staining alone, irrespective of nucleic staining (Method C), (2) independent nucleic staining alone, irrespective of cytoplasmic staining (Method N), and (3) mixed cytoplasmic with nucleic expression, using mean of the sum of cytoplasmic score plus nucleic score (Method Mean of C plus N). Of the 14 ECA and 24 EMA tissue samples in this study, we found that only 2 (Method N as well as Method Mean of C plus N) out of the total 3 scoring methods showed significant frequency differences (p < 0.05), whereas the third scoring method (Method C) did not show a significant difference (p > 0.05) in distinguishing between ECAs and EMAs. (Table 1) We can not completely yet rule out the possibility of the indigenous heterogeneity within individual tumors, leading to different p16^INK4a ^expression patterns in various areas within the same tissue samples, because of the limited number of cases (14 ECA and 24 EMA tissues) and limited core size (1.5 mm) of the tumor specimens in TMA. However, our data showed that cytoplasmic p16^INK4a ^expression correlated significantly with nucleic p16^INK4a ^expression (p = 0.007) in EMAs, but not to do so (p = 0.663) in ECA. In short, cytoplasmic and nucleic staining correlates closely in EMAs, but do not in ECAs.

For the p16^INK4a^-marker characteristics and test effectiveness of ECA and EMA discrimination, the goal is to minimize the chance or probability of false positive and false negative results, and to maximize the probability of true positive and true negative results. According to our data, one method based on C (Method C) does not show significant frequency difference in making distinction between ECAs and EMAs (p = 0.245). The sensitivity of Method C was 35.7%, indicating a high false negative rate, whereas, the specificity of Method C is 81.0%, indicating a favorable low false positive rate. Both the negative predictive value (69.0%) and the positive predictive value (55.6%) do not provide valuable information. However, the scoring of p16^INK4a ^expression using the other 2 mechanisms, including Method N and Method Mean of C plus N, shows significant frequency differences in making distinction between ECAs and EMAs (p < 0.05). The highest sensitivity is 78.6% using Method N, the highest specificity is 85.7% using Method Mean of C plus N, the highest negative predictive value is 85% using Method N, whereas the highest positive predictive value is 77.0% using Method Mean of C plus N. Notably, Method Mean of C plus N has the highest overall accuracy (80%).

In summary, of the three p16^INK4a^-scoring mechanisms, Method N and Method Mean of C plus N are useful in distinguishing these two gynecologic adenocarcinomas (ECA *vs. *EMA), whereas Method C is not. Using the Method Mean of C plus N in p16^INK4a^-marker IHC assessments, deserves the most favorable test effectiveness and performance of all, which may not only assist physicians in making adequate diagnostic distinction between ECAs and EMAs, but also help individual patients by appropriate treatment options. Despite the finite number of cases, our data provides significant and valuable reference to verify that p16^INK4a ^with appropriate scoring mechanisms can be applied in designing the appropriately diagnostic multi-marker panels in distinguishing between ECAs and EMAs.

## Conclusion

Although careful gross and histologic examinations usually allows a confidant distinction between ECAs and EMAs, diagnostic dilemma may occur when tumor is localized in both endometrial and endocervical biopsies, histomorphologic overlaps and preoperative imaging studies may also confuse in establishing the site of origin. True diagnosis may require the assistance of ancillary IHC stains. The p16^INK4a ^marker tends to be positively and diffusely expressed in ECAs, but tends to be negatively or focally expressed in EMAs. However, there is still a lack of optimized consensus or standard for p16^INK4a ^IHC scoring mechanisms. According to the scientific results in this study, we found that Method Mean of C plus N and Method N can help to distinguish between ECAs and EMAs, but Method C is of no use to do so. Based on the data characteristics and test effectiveness, Method Mean of C plus N is the most promising score-calculating means among the three p16^INK4a ^IHC scoring mechanisms in diagnostic distinction between these two gynecologic malignancies (ECAs *vs. *EMAs).

## List of abbreviations used

ECAs: Endocervical adenocarcinomas; EMAs: Endometrial adenocarcinomas; p16: p16^INK4a^; IHC: Immunohistochemistry, Method C: Independent cytoplasmic staining alone; Method N: Independent nucleic staining alone; Method Mean of C plus N: Mean of cytoplasmic score plus nucleic score.

## Competing interests

The authors declare that they have no competing interests.

## Authors' contributions

CPH, LFK, CLK performed experiments and wrote the manuscript. YWC provided monoclonal anti-mouse p16^INK4a ^antibody (F12, sc-1661, Santa Cruz) and carried out the immunohistochemical stains. MYL performed the statistical analysis. KCC, JDH participated in its design and coordination. TSW, AR executed an important part of the study and edited the draft manuscript. All authors read and approved the final manuscript.
